# Comparison of efficiency and safety of rivaroxaban, apixaban and enoxaparin for thromboprophylaxis after arthroplastic surgery: a meta-analysis

**DOI:** 10.1042/BSR20180423

**Published:** 2018-11-14

**Authors:** Zhi Yu, Ping Shan, Xiaoxia Yang, Xin-jiang Lou

**Affiliations:** Department of Vascular Surgery, Second Affiliated Hospital of Zhejiang Chinese Medical University, Hangzhou 310005, China

**Keywords:** Apixaban, clinical outcomes, deep venous thrombosis, Enoxaparin, Rivaroxaban, thromboprophylaxis

## Abstract

Objective: To compare the efficacy and safety of rivaroxaban, apixaban and enoxaparin for thromboprophylaxis after arthroplastic surgery. Methods: We conducted a meta-analysis containing a wide range of randomized controlled trials about efficiency and safety of rivaroxaban, apixaban and enoxaparin for thromboprophylaxis after arthroplastic surgery in the recent decade from January 2006 to June 2018. The present study separately analyzed the following key components: the different efficiency and safety for rivaroxaban and enoxaparin; apixaban and enoxaparin; and enoxaparin and other new developed anticoagulants. Results: Sixteen studies containing 58885 patients were included. In results of efficacy outcomes, total events occurred in 4.89% patients of rivaroxaban group and 9.55% patients of the control group; however, no significant difference was observed in apixaban groups of their efficacy outcomes. Primary events didn’t show significant difference when comparing apixaban with the control or comparing enoxaparin with the control. In analysis of safety outcomes, bleeding events occurred in 3.41% patients of rivaroxaban group compared with 2.84% patients of the control groups; bleeding events in apixaban groups were 4.09% compared with the control groups 4.64%. Bleeding events occurred in 3.51% patients of enoxaparin group, slightly lower than 5.82% of the control group. Conclusion: Direct oral anticoagulant, rivaroxaban might have better efficacy outcomes in thromboprophylaxis after arthroplastic surgery; however, apixaban showed no significantly different efficacy outcomes compared with enoxaparin, and enoxaparin may have equal or even better safety outcomes compared with direct oral anticoagulants.

## Introduction

Venous thromboembolism (VTE), including deep-vein thrombosis (DVT) and pulmonary embolism (PE), frequently occurs after arthroplastic surgery, such as total hip arthroplasty (THA) or total knee arthroplasty (TKA) [1,2]. According to clinic data, patients undergoing TKA are at higher risk for developing DVT; while the rate of symptomatic DVT is higher after THA [3,4]. Published estimates showed that VTE affects more than 600,000 people every year in the US [5]. In Asia, a large epidemiological study demonstrated that the incidence of DVT was 41% if antithrombotic drugs were not used after THA or TKA surgery [6]. A high risk of recurrent VTE, including fatal and non-fatal PE, exists in patients with symptomatic DVT and may persist for years [7]. Thus, to prevent DVT after THA, TKA remains to be the key component of arthroplastic surgery prognosis.

Generally, regular use of antithrombotic drugs is suggested for the prevention of DVT in patients who have undergone THA or TKA [8]. It is reported that the incidence of VTE may be reduced to 50% when antithrombotic drugs are used during THA or TKA [9]. At present, recommended drugs for VTE include unfractionated heparin [10] and low-molecular-weight heparin such as enoxaparin, fondaparinux and vitamin K antagonists, like warfarin [11]. Despite being widely used for years, traditional treatments are proved to have numerous limitations, such as parenteral administration, a slow onset of action, regular coagulation monitoring and numerous drug and food interactions [12,13].

In the recent decade, several new drugs are developed, such as direct inhibitors of thrombin (dabigatran) and factor Xa (rivaroxaban, apixaban) [14]. These drugs have a stable and predictive pharmacokinetic and pharmacodynamic profile compared with the old ones, so they have attracted lots of studies to evaluate their clinic conditions [15]. One serious complication of frequently used antithrombotic drugs (such as warfarin or heparin) is hemorrhage [16]. Studies also showed that rivaroxaban has an increased risk of bleeding complications compared with enoxaparin [17].

Since both rivaroxaban and apixaban are typical new drugs that were widely used in these years, we also wanted to provide more evidences for their clinical efficiency and safety from our own perspectives. What’s more, though several studies have already analyzed the efficacy and safety of rivaroxaban and apixaban after THA and TKA [18–20], most of them focused on the clinic trials including comparison studies, and since both rivaroxaban and apixaban were approved in recent years, the involved studies of existed analysis papers were always few. In the present study, we aimed to conduct a meta-analysis to analyze the efficiency and safety of rivaroxaban and apixaban, using one of the most widely used traditional anticoagulant enoxaparin as a comparison. The present study separately analyzed the following key components: the different efficiency and safety for rivaroxaban and enoxaparin; apixaban and enoxaparin; and enoxaparin and other new developed anticoagulants, including a larger range of clinic trials in the last decade to show their respective efficacy and safety in preventing venous thromboembolism after THA and TKA.

## Methods

The present study was approved by the medical ethics committee of Xinhua Hospital of Zhejiang Province

### Study selection criteria

Before searching the literature, criteria were set for articles of rivaroxaban, apixaban and enoxaparin in thromboprophylaxis after arthroplastic surgery. The following standards were required for the included studies: (1) it was a randomized controlled trial; (2) patients of all ages undergoing total hip or knee replacement were involved; (3) the efficacy and safety of rivaroxaban, apixaban or enoxaparin were studied; (4) studies only published in English; (5) studies published during January 2006 to 30 June 2018. Trials with a blinded or unblinded design were both included; control groups were included in each study but the control drugs were not specified.

### Literature search

Articles published from 1 January 2006 to 30 June 2018 were searched from PubMed, EMBASE, Elsevier, Springer and Google scholar. The search terms were combination of the following keywords: rivaroxaban, apixaban, enoxaparin, deep vein thrombosis, total hip arthroplasty and total knee arthroplasty. A manual search was also conducted through searching the reference lists of relevant articles to expand the included studies.

### Data collection and extraction

Two independent observers (Zhi Yu and Ping Shan) reviewed abstracts for qualification examination according to the predefined criteria. Selected papers were then retrieved, evaluated for their eligibility, and relevant data were extracted by the two observers independently. A third observer (Xiaoxia Yang) was consulted when disagreements occurred. The following items were extracted according to a fixed protocol: author, year of publication, study type, original study population, number of cases and drug tested. The primary efficacy outcome of this meta-analysis was a composite of DVT, non-fatal pulmonary embolism and all-cause mortality. The primary safety outcome of the meta-analysis was bleeding event, defined as major bleeding that was fatal, occurred in a critical organ or needed re-operation and clinically relevant non-major bleeding. When multi-dose was used in a study, cases in all doses were considered as the integrated result.

### Statistical methods

Pooled data were analyzed using the Review Manager 5.3 (The Nordic Cochrane Centre; Copenhagen, Denmark). The outcomes were assessed using random effects models and statistical heterogeneity was evaluated using the *I*^2^ statistic. The odds ratio (OR) were calculated for each outcome with 95% confidence intervals (CI). A *P*-value of 0.05 or less was considered statistically significant. The main analysis was on an intention to treat basis. Publication bias was assessed using Review Manager 5.3 by analysis of Cochrane Collaboration’s risk of bias.

## Results

### Study collection and characteristics

**Figure 1:**
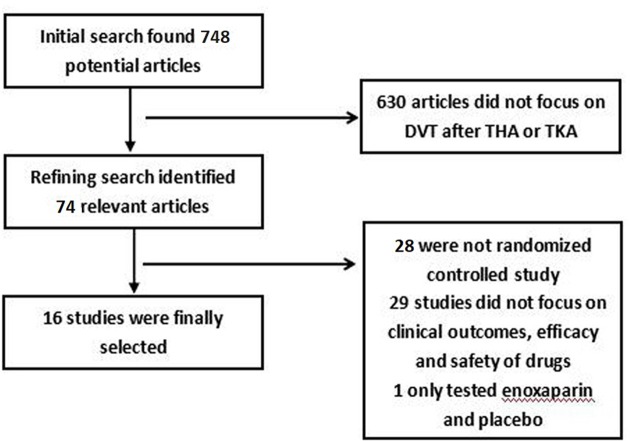
Detailed search results

Initial search identified 748 reference articles, in which 74 relevant articles were selected and reviewed. After reviewing the abstract, 28 references were excluded. And after evaluation of the full texts, 28 references were further excluded. Among the rest 17 references, 1 only tested enoxaparin and placebo, thus data were finally extracted from 16 studies [21–36]. All selected studies are in accordance with the inclusion criteria. See Figure 1 for detailed search results.

As shown in Table 1, 16 studies were included in this meta-analysis, from 2006 to 2016. All studies were randomized controlled studies and 12 of them were double-blind trials. Among the studies, rivaroxaban was tested in 8 studies, apixaban was tested in 3 studies and enoxaparin was tested in all the 16 studies. The whole studies included 58,885 patients.

**Table 1:** Summary table of the meta-analysis

Authors	Year	Study type	Cases	Tested	Controlled	Days of treatment (days)
Özler [21]	2015	Non-blinded, randomized controlled study	180, body weight >50 kg and age ≥18 years	Rivaroxaban 1 × 10 mg (or 1 × 220 mg Dabigatran) during the outpatient period for a total of 10 days after TKA and 30 days after THA	Enoxaparin 2 × 0.3 ml during the hospital stay and 1 × 0.4 ml enoxaparin during the outpatient period	10–30
Rosencher [22]	2013	Randomized controlled study	12,500, body weight >50 kg and age ≥18 years	Rivaroxaban 10 mg once daily started 6–8 h after surgery	Enoxaparin 30 mg twice daily starting 12–24 h after wound closure for 10–14 days; or 40 mg once daily starting 12 h before surgery	10–14
Eriksson [23]	2012	Randomized, double-blind study	12,110, body weight >50 kg and age ≥18 years	Rivaroxaban 10 mg once daily (od) starting 6–8 h after surgery	Enoxaparin 40 mg od starting 12 h before surgery; or 30 mg twice daily starting 12–24 h after adequate hemostasis was achieved	10–39
Turpie [24]	2009	Randomized, double-blind study	3148, body weight >50 kg and age ≥18 years	Rivaroxaban 10 mg once daily, beginning 6–8 h after surgery	Enoxaparin 30 mg every 12 h, starting 12–24 h after surgery	11–15
Kakkar [25]	2008	Randomized, double-blind study	1049, body weight >50 kg and age ≥18 years	Rivaroxaban 10 mg once daily 6–8 h after wound closure	Enoxaparin 40 mg once daily 12 h before surgery and restarted 6–8 h after wound closure	10–39
Eriksson [26]	2007	Randomized, open-label, study	625, body weight >50 kg and age ≥18 years	Rivaroxaban 2.5, 5, 10, 20 and 30 mg twice daily [bid] or 30 mg once daily [od] starting 6–8 h after surgery	Enoxaparin 40 mg od starting the evening before surgery	5–9
Eriksson [27]	2008	Randomized, double-blind study	4433, body weight >50 kg and age ≥18 years	Rivaroxaban 10 mg dose beginning after surgery	Enoxaparin 20 mg administered subcutaneously once daily beginning the evening before surgery	36
Lassen [28]	2008	Randomized, double-blind study	2531, body weight >50 kg and age ≥18 years	Rivaroxaban, 10 mg once daily, beginning 6–8 h after surgery	Enoxaparin 40 mg once daily, beginning 12 h before surgery	13–17
Lassen [29]	2010	Randomized, double-blind study	3057, body weight >50 kg and age ≥18 years	Apixaban 2.5 mg twice daily 12–24 h after wound closure	Enoxaparin 40 mg once daily 12 h before surgery	10–14
Lassen [30]	2010	Randomized, double-blind study	5407, body weight >50 kg and age ≥18 years	Apixaban 2.5 mg orally twice daily 12–24 h after closure of the surgical wound	Enoxaparin 40 mg subcutaneously every 24 h, 12 h before surgery	35
Lassen [31]	2009	Randomized, double-blind study	3608, body weight >50 kg and age ≥ 18 years	Apixaban 2.5 mg orally twice daily 12–24 h after surgery	Enoxaparin 30 mg subcutaneously every 12 h, 12–24 h after surgery	10–14
Fuji [32]	2014	Randomized, double-blind study	716, body weight >50 kg and age ≥18 years	Enoxaparin 2000 IU (equivalent to 20 mg) subcutaneously twice daily beginning 24–36 h postsurgery	Edoxaban 30 mg once daily beginning 6–24 h postsurgery	11–14
Eriksson [33]	2011	Randomized, double-blind study	2055, body weight >50 kg and age ≥18 years	Enoxaparin 40 mg once daily, starting the evening before surgery	Dabigatran 220 or 150 mg once daily, starting with a half-dose 1–4 h after surgery	28–35
Ginsberg [34]	2009	Randomized, double-blind study	1896, body weight >50 kg and age ≥18 years	Enoxaparin 30 mg SC BID after surgery	Dabigatran etexilate 220 or 150 mg once daily after surgery	12–15
Eriksson [35]	2007	Randomized, double-blind study	2076, body weight >40 kg and age ≥18 years	Enoxaparin 40 mg once-daily, starting the evening before surgery	Dabigatran etexilate, 150 or 220 mg once-daily, starting with a half-dose 1–4 h after surgery	6–10
Eriksson [36]	2007	Randomized, double-blind study	3494, body weight >50 kg and age ≥18 years	Enoxaparin 40 mg once daily, starting the evening before surgery	Dabigatran etexilate 220 or 150 mg once daily, starting with a half-dose 1–4 h after surgery	28–35

### Pooled analysis of efficacy outcomes

**Figure 2:**
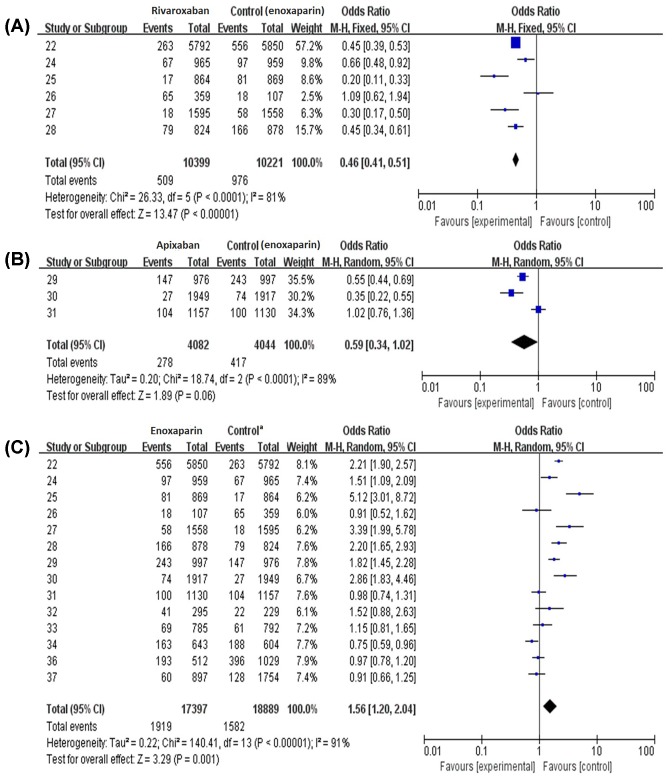
Efficacy outcomes of rivaroxaban, apixaban and enoxaparin in DVT of patients after TKA and THA (**A**) Efficacy outcomes of rivaroxaban. (**B**) Efficacy outcomes of apixaban. (**C**) Efficacy outcomes of enoxaparin. ^a^In efficacy of enoxaparin, the control groups included drugs of rivaroxaban, dabigatran, apixaban, edoxaban and dabigatran etexilate.

Two studies didn’t show useful primary efficacy data [21,23] but only the safety data, thus they were only included in the safety analysis. Results of efficacy outcomes of rivaroxaban, apixaban and enoxaparin were shown in Figure 2A–C. The primary efficacy outcome of this meta-analysis was a composite of DVT, non-fatal pulmonary embolism and all-cause mortality. In the result of primary efficacy outcome of rivaroxaban, all control groups were treated with enoxaparin. Total events occurred in 4.89% (509/10399) patients in rivaroxaban group compared with 9.55% (976/10221) patients in the control group, indicating rivaroxaban had a trend to decrease the events (RR 0.46, 95% CI 0.41-0.51), *P*<0.0001 (Figure 2A). Efficacy result of apixaban was shown in Figure 2B, in this section all control groups were also enoxaparin. The efficacy outcome of apixaban groups didn’t show significant difference compared with the control groups (RR 0.59, 95% CI 0.34-1.02), *P*>0.05. In efficacy of enoxaparin shown in Figure 2C, 14 studies were included, containing a total of 36,286 patients. The control groups included drugs of rivaroxaban, dabigatran, apixaban, edoxaban and dabigatran etexilate, all new anticoagulants. Results showed that events occurred in 11.03% patients (1919/17,397) of enoxaparin groups compared with 8.38% patients (1582/18,889) of the control groups, indicating that enoxaparin had a trend to increase the events (RR 1.56, 95% CI 1.20-2.04), *P*<0.05.

### Pooled analysis of safety outcomes

**Figure 3:**
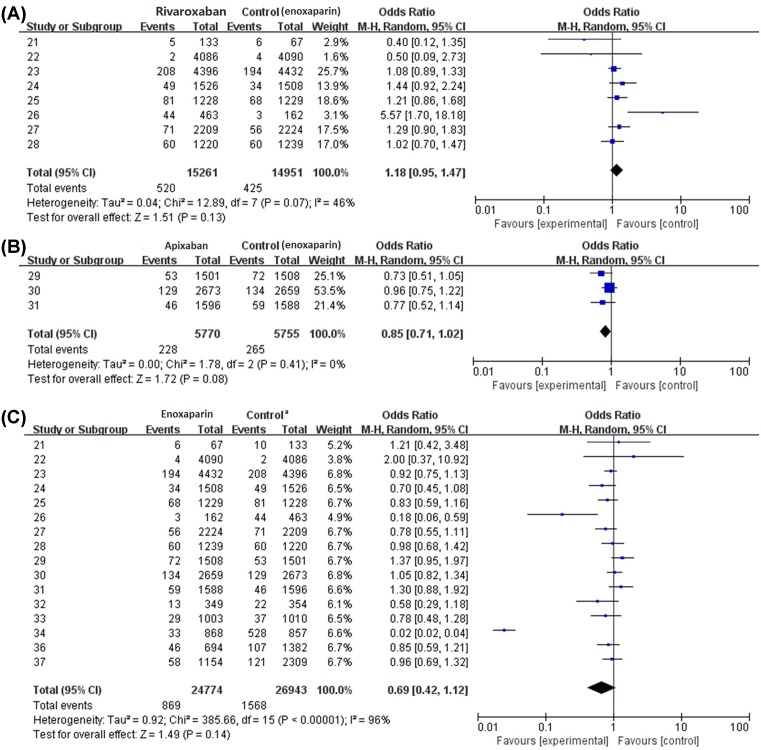
Safety outcomes of rivaroxaban, apixaban and enoxaparin in DVT of patients after TKA and THA (**A**) Safety outcomes of rivaroxaban. (**B**) Safety outcomes of apixaban. (**C**) Safety outcomes of enoxaparin. ^a^In efficacy of enoxaparin, the control groups included drugs of rivaroxaban, dabigatran, apixaban, edoxaban and dabigatran etexilate.

In analysis of safety outcomes, all 16 studies were involved. The primary safety outcome of the meta-analysis was bleeding event, defined as major bleeding and clinically relevant non-major bleeding. Figure 3A showed the results of safety outcomes of rivaroxaban, bleeding events occurred in 3.41% patients (520/15,261) of rivaroxaban groups compared with 2.84% patients (425/14,951) of the control groups, which were all enoxaparin. This result suggested that rivaroxaban may have a trend to increase the bleeding events compared with enoxaparin; however, the difference is not significant (RR 1.18, 95% CI 0.95–1.47), *P*>0.05. Similar results were obtained in apixaban groups 4.09% (228/5570) compared with the control groups 4.64 (265/5755) that were all enoxaparin, indicating that no significant difference was observed in bleeding events of the two drugs (RR 0.85, 95% CI 0.71–1.02), *P*>0.05 (Figure 3B). In results of safety outcomes of enoxaparin, bleeding events of enoxaparin groups 3.51% (869/24,774) were slightly lower than the control groups 5.82% (1568/26,943) that were all new anticoagulants, indicating enoxaparin had a trend to decrease the bleeding events compared with the new anticoagulants. However, the effect was not significant (RR 0.69, 95% CI 0.42–1.12), *P*>0.05 (Figure 3C).

### Assessment of bias

**Figure 4:**
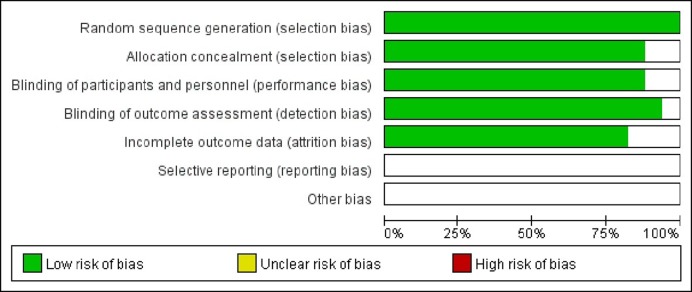
Publication bias by Review Manager 5.3 using Cochrane Collaboration’s risk of bias for all the studies included in the meta-analysis

The publication bias was examined using analysis of Cochrane Collaboration’s risk of bias by Review Manager 5.3 (Figure 4). No significant publication bias was observed.

## Discussion

In the present study, we separately analyzed the different efficiency and safety for rivaroxaban and enoxaparin; apixaban and enoxaparin; and enoxaparin and other new developed anticoagulants, using a larger range of clinic trials in the last decade to show their respective efficacy and safety in preventing venous thromboembolism after THA and TKA. The study covered the largest number of researches from January 2006 to June 2018 and results showed that only rivaroxaban showed significantly better efficiency than enoxaparin; however enoxaparin showed equal safety compared with other new oral anticoagulants.

Several studies have shown that rivaroxaban and apixaban demonstrate better clinical outcomes compared with enoxaparin. However mainly due to the recent approval of ivaroxaban and apixaban, studies involved in these analysis were always few. In an analysis conducted by Nieto et al. [18], 10 studies were involved: 3 tested rivaroxaban, 3 tested apixaban, 4 tested other direct inhibitors of thrombin (dabigatran), and all studies used enoxaparin as a control. In this meta-analysis, we wanted to extend the research range to include more new studies conducted in recent years and focus our eyes on inhibitors of factor Xa (rivaroxaban, apixaban) compared with enoxaparin. What’s more, we also investigated the efficacy and safety of enoxaparin compared with new anticoagulants including not only rivaroxaban and apixaban, but also drugs like dabigatran, dabigatran etexilate and edoxaban that were approved recently.

The analysis finally chose 16 randomized controlled studies with a total of 58,885 patients included in this meta-analysis. Results of efficacy outcomes of rivaroxaban, apixaban and enoxaparin demonstrated that rivaroxaban had a trend to decrease the primary clinical events (RR 0.46, 95% CI 0.41–0.51) compared with enoxaparin. And the efficacy outcome of apixaban groups didn’t show significant difference compared with enoxaparin (RR 0.59, 95% CI 0.34–1.02). These results were similar to other studies [18,37]. Then we compared enoxaparin with several new anticoagulants developed in the recent decade, containing a total of 36,286 patients, and observed a consistent result showing that enoxaparin had a trend to increase the events (RR 1.56, 95% CI 1.20–2.04).

Some studies found that compared with enoxaparin, apixaban and rivaroxaban could decrease DVT but not bleeding after THA or TKA [38,39]. In this meta-analysis, we obtained similar results and we found no significant difference was observed in bleeding events of apixaban and enoxaparin (RR 0.85, 95% CI 0.71–1.02). However, rivaroxaban may have a trend to increase the bleeding events compared with enoxaparin, though the difference is not significant (RR 1.18, 95% CI 0.95–1.47). This result is also in consistent with other previous studies that demonstrated rivaroxaban may increase bleeding [17]. What’s more, enoxaparin may have a better effect on decreasing the bleeding event after THA and TKA compared with new anticoagulants, though the effect was not significant (RR 0.69, 95% CI 0.42–1.12). All these results could point to the conclusion that rivaroxaban may have better efficacy outcomes in preventing venous thrombosis after THA or TKA, but enoxaparin may have equal or even better safety outcomes.

There were also several limitations that must be considered in the present study. First, though we wanted to conduct a wide range study to contain all related studies in the recent decade, the number of newer conducted studies was still not much. Second, we tried to obtain as bigger sample size as we can to get a more general result, thus we ignore some of the detailed difference such as the different doses used in each study and combined them as an integrated outcome. Also we noticed that the drugs involved in the study were used not only in English speaking countries, but also in many non-English speaking countries that should be paid attention to. At last, efficacy and safety of these drugs for children should also be further considered.

In conclusion, we conducted a meta-analysis containing a wide range of studies focusing on rivaroxaban, apixaban and enoxaparin for thromboprophylaxis after arthroplastic surgery in the recent decade. Study showed that only rivaroxaban might have better efficacy outcomes in thromboprophylaxis after THA or TKA, but enoxaparin might have equal or even better safety outcomes.

## Abbreviations

DVT: deep-vein thrombosis
PE: pulmonary embolism
THA: total hip arthroplasty
TKA: total knee arthroplasty
VTE: venous thromboembolism
